# Risk factors for postoperative hypokalemia in patients undergoing endoscopic pituitary adenoma resection: a retrospective cohort study

**DOI:** 10.7717/peerj.18536

**Published:** 2024-11-18

**Authors:** Maoxiang Li, Changhong Mo, Sifan Yan, Ruijing Zhao, Weijian Luo, Lu Yang, Hao Wang, Jiliang Hu

**Affiliations:** 1 The Second Clinical Medical College of Jinan University, Shenzhen, Guangdong, China; 2 Department of Neurosurgery, The Sixth Affiliated Hospital, School of Medicine, South China University of Technology, Foshan, Guangdong, China; 3 The Department of Neurosurgery, Shenzhen People’s Hospital, Shenzhen, Guangdong, China; 4 Guangdong Engineering Technological Research Center for nervous anatomy and Related Clinical Applications, Shenzhen, Guangdong, China; 5 The First Affiliated Hospital of the Southern University of Science and Technology, Shenzhen, Guangdong, China

**Keywords:** Endoscopic transsphenoidal surgery, Hypokalemia, Pituitary adenoma, Postoperative complications

## Abstract

**Background:**

Currently, endoscopic transsphenoidal surgery is the primary approach for treating pituitary tumors. While endoscopic surgery offers numerous advantages, it also comes with a series of potential surgical complications. Postoperative hypokalemia is a common complication, with mild cases presenting with atypical symptoms such as dizziness, headache, fatigue, and constipation, while severe cases can lead to arrhythmias, rhabdomyolysis, and even death. Therefore, early identification of risk factors for postoperative hypokalemia is crucial. This study aims to analyze the risk factors for hypokalemia after endoscopic pituitary tumor resection.

**Methods and Materials:**

This study included 168 patients who underwent endoscopic resection of pituitary tumors between 2019 and 2023. Patients were divided into hypokalemia group and non-hypokalemia group based on whether their postoperative serum potassium concentration was less than 3.0 mEq/L. Identifying independent risk factors through binary logistic regression analysis.

**Results:**

Among the 168 patients, 18 (10.7%) cases experienced postoperative hypokalemia, with the majority occurring on the fourth day after surgery. The majority of patients did not exhibit clinical symptoms related to hypokalemia. The binary logistic regression analysis revealed that age (OR 1.09; 95% *CI* [1.03–1.15]; *P* = 0.001) and postoperative hypoalbuminemia on the first day (OR, 4.35; 95% *CI* [1.38–13.75]; *P* = 0.012) were associated with postoperative hypokalemia.

**Conclusions:**

Patients aged ≥50 years and those presenting with hypoalbuminemia on the first postoperative day were more likely to develop postoperative hypokalemia. Therefore, electrolyte monitoring should be enhanced in such patients postoperatively, especially to actively prevent hypokalemia on the 4th–5th postoperative day.

## Introduction

Pituitary tumors are common intracranial tumors, with the majority being benign, but a minority can be invasive. Clinical symptoms are usually related to the degree of compression on surrounding structures by the pituitary tumor and the disruption of pituitary hormone balance, such as headaches, dizziness, visual field defects, and decreased pituitary endocrine function (Molitch, 2008; Melmed, 2015). Excess or deficiency of pituitary hormones is associated with patient mortality rates, such as acromegaly, Cushing’s disease, and hypopituitarism crises (Pappachan et al., 2015). Endoscopic endonasal pituitary tumor resection, using a transnasal endoscope approach, has the advantage of clear illumination and realistic observation. However, there are also many surgical complications. Among them, postoperative hypokalemia is a common electrolyte disorder, with symptoms including dizziness, headaches, nausea, vomiting, and weakness. Severe hypokalemia can lead to arrhythmias, respiratory failure, rhabdomyolysis, and even death (Gennari, 1998). Existing research indicates a correlation between postoperative hypokalemia in patients with pituitary adenomas and increased mortality rates (You et al., 2017). Although substantial attention has been given to postoperative electrolyte imbalances in pituitary tumors, such as hyponatremia, the specific issue of hypokalemia has not been as thoroughly investigated. Pioneering studies by You et al. (2017) have examined the relationship between ACTH-secreting pituitary adenomas and postoperative hypokalemia. Concurrently, Li et al. (2021) have developed predictive models for hypokalemia risk using a preoperative index-based nomogram. Therefore, hypokalemia is a postoperative complication that requires attention. Early detection and intervention of hypokalemia not only can shorten hospital stay and reduce medical costs, but also can decrease the occurrence of various complications.

This study aims to analyze the risk factors for postoperative hypokalemia following endoscopic endonasal pituitary tumor resection, in order to promptly identify factors leading to postoperative hypokalemia.

## Materials and Methods

### Study population

We retrospectively analyzed data from 168 patients who underwent endoscopic pituitary tumor resection and were pathologically confirmed to have pituitary adenomas from 2019 to 2023. All surgeries were performed by two experienced neurosurgeons. This study has been approved by the Ethics Review Committee with the ethical approval number of LL-KY-2023055 on May 5th, 2023. The study involved anonymous data and was exempt from informed consent. Patients were divided into hypokalemia group and non-hypokalemia group based on postoperative serum potassium concentration being less than 3.0.

### Perioperative assessment

All patients involved in the study underwent endoscopic pituitary tumor resection and were transferred to the neurosurgical intensive care unit for treatment and monitoring postoperatively. We focused on monitoring patients’ blood pressure, heart rate, pulse, respiratory rate, level of consciousness, visual acuity, urine output, and signs of cerebrospinal fluid leakage. All patients fasting on the day of operation and need to receive parenteral nutrition support to supplement the physiological requirements of potassium. Stable patients resumed regular diet on the first day after surgery. Most patients underwent potassium testing before surgery, on the first day postoperatively, and subsequently every 2 days until discharge. Some patients had potassium levels tested before surgery, on the first and second day postoperatively, followed by routine testing every 2 days until discharge. In case of symptomatic patients, electrolyte levels were immediately checked. Patients with preoperative hyponatremia or hypokalemia had their sodium and potassium levels normalized before surgery. Patients with preexisting pituitary insufficiency received replacement therapy. Most patients with hypokalemia received oral potassium supplementation and were closely monitored until levels returned to normal. For severe hypokalemia, intravenous potassium supplementation was required. Patients receiving intravenous potassium needed close monitoring of blood pressure, heart rate, electrocardiogram, among other parameters. Similarly, patients with postoperative pituitary insufficiency received replacement therapy. Desmopressin was used for patients with diabetes insipidus. Within the first week after discharge, patients needed to follow up at the neurosurgery or endocrinology outpatient clinic for hormone and electrolyte monitoring.

### Data collection

We gathered baseline information for all patients, including age, gender, BMI, history of hypertension, history of diabetes, among others. All patients underwent imaging studies, hematological and biochemical tests, as well as endocrine hormone examinations preoperatively. Through head MRI imaging, tumor Knosp classification, tumor height, maximum diameter, volume, presence of intracapsular cysts or hemorrhage, and optic chiasm compression were determined. The serum levels of potassium, sodium, and calcium were evaluated through biochemical tests. Pituitary function was assessed through endocrine hormone examinations. Using coronal position images from head MRI, the line connecting the tumor to the internal carotid artery cavernous sinus segment and the upper clivus vasculature inner edge was measured for Knosp classification of the tumor. The distribution of Knosp classifications were as follows: Grade 0 had 29 patients (17.3%), Grade 1 had 39 patients (23.2%), Grade 2 had 38 patients (22.6%), Grade 3 had 37 patients (22.0%), and Grade 4 had 25 patients (14.9%). Hyponatremia was categorized as mild (131–135 mEq/L), moderate (125–130 mEq/L), and severe (<125 mEq/L). Hypokalemia was categorized as mild (3.0–3.5 mEq/L), moderate (2.5–3.0 mEq/L), and severe (<2.5 mEq/L). Patients presenting with preoperative hypokalemia or hyponatremia were treated accordingly. Definition of hypopituitarism is consistent with a previous study (Mo et al., 2024). Post-operative hypophysial-thyroid axis/hypophysial-gonadal axis/cortisol dysfunction is defined as a lower than normal level of any hormone above the endocrine hormone test on the first day after surgery. Postoperative hypoalbuminemia was defined as a serum albumin concentration <35 g/L as measured by blood biochemistry on the first day after surgery. At our center, serum albumin levels are measured by collecting blood from the patient’s elbow vein at 8 am on the morning after surgery for testing. Postoperative diabetes insipidus (DI) was defined as postoperative urine output ≥300 ml/h for at least 2 h. Research suggests that symptoms typically present when serum potassium falls below 3.0 mEq/L (Kardalas et al., 2018). Therefore, patients with postoperative serum potassium concentrations <3.0 mEq/L were included in the postoperative hypokalemia group. The timing of hypokalemia occurrence was based on the available test results, although some patients may have developed hypokalemia before testing.

### Statistical analysis

We performed statistical analysis on the data using SPSS version 26.0 (IBM, Armonk, New York, USA). For normally distributed variables, independent sample t-tests were conducted, with results expressed as mean ± standard deviation. For non-normally distributed variables, the Mann-Whitney U test was employed, and results were presented using median (*p*25, *p*75). Categorical variables were analyzed using the chi-square test, except for variables where any cell value in the contingency table was ≤5, in which case Fisher’s exact test was used, and results were presented as counts (percentages).

**Figure 1:**
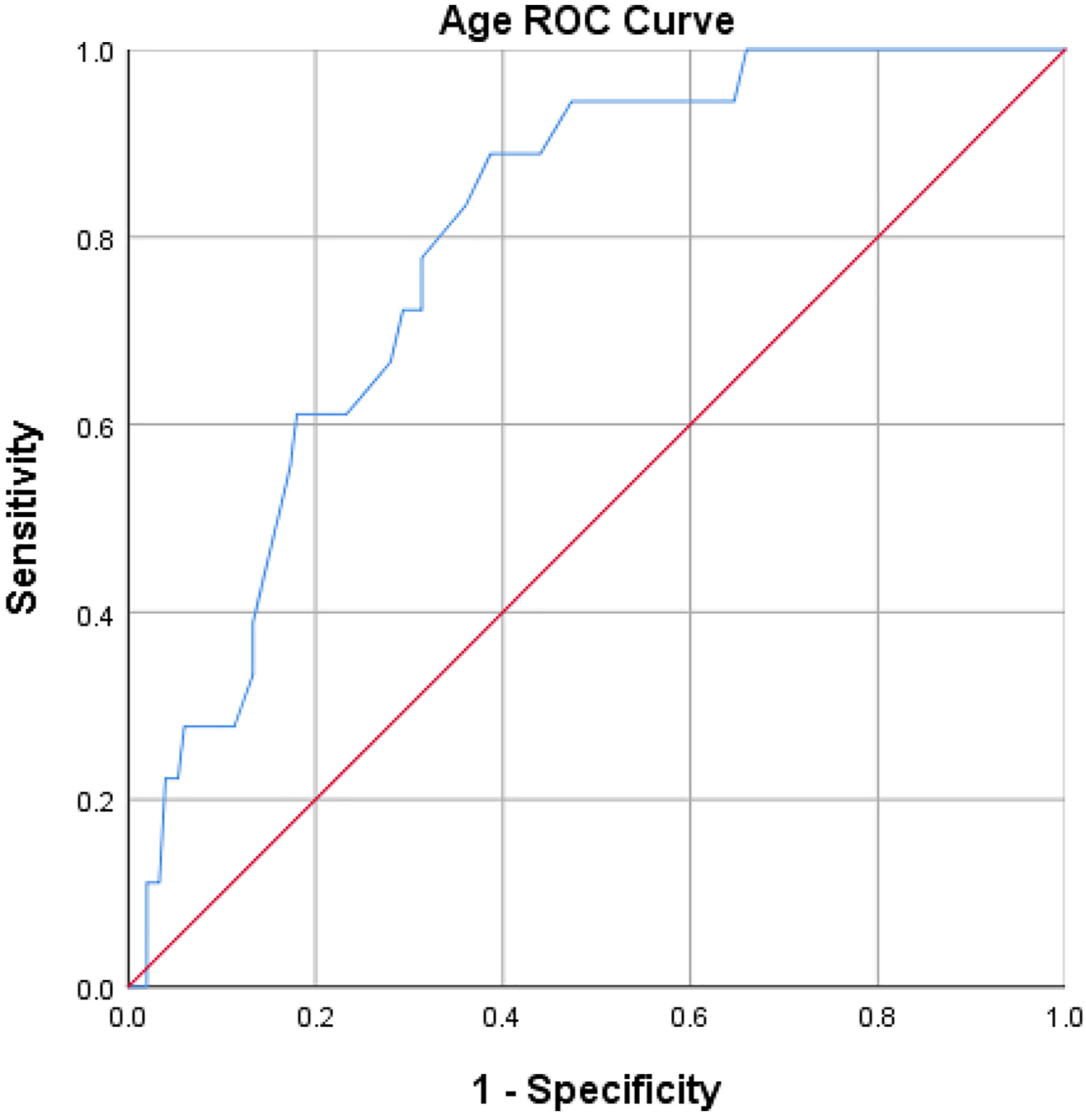
ROC curve analysis of age. The result of the ROC curve analysis showed that the optimal cut-off value for age was 50, with a sensitivity of 88.9% and a specificity of 61.3%. The area under the curve (AUC) was 0.792 (95% *CI* [0.70–0.88]).

Factors with *p* ≤ 0.1 in the univariate analysis were included in binary logistic regression analysis, with factors having *p* < 0.05 defined as statistically significant. Receiver operating characteristic (ROC) curves were plotted for age to determine the optimal threshold value and the area under the curve (AUC). The optimal cutoff value for age was determined as 50 years through the construction of an age ROC curve (sensitivity 88.9%, specificity 61.3%), with an AUC of 0.792, 95% *CI* [0.70–0.88] (Fig. 1).

## Results

### Patients’ characteristics

In the entire cohort, there were 74 (44.1%) males and 94 (56.0%) females. The median age was 46.8 years (range 19 to 77 years). 55 (32.7%) cases had hypertension; 25 (14.9%) cases had diabetes mellitus. Eighteen (10.7%) cases experienced postoperative hypokalemia, with 5 (27.8%) cases presenting symptoms like dizziness, headache, nausea, vomiting, and respiratory difficulty. Cerebrospinal fluid leakage occurred in 33 (19.6%) cases during surgery. Thirty-one (18.5%) cases of hypoproteinemia on the first day after operation. Postoperative diabetes insipidus was observed in 80 (47.6%) cases. Based on postoperative pathology, 32 (19.1%) cases had non-functional adenomas, 19 (11.3%) cases had GH-secreting adenomas, 33 (19.6%) cases had PRL-secreting adenomas, 41 (24.4%) cases had FSH-secreting adenomas, 29 (17.3%) cases had ACTH-secreting adenomas, four (2.4%) cases had TSH-secreting adenomas, and 10 (6.0%) cases had plurihormonal adenomas (Table 1).

**Table 1:** Clinical characteristics and auxiliary examinations.

Factors	Total (*n* = 168)	Hypokalemia (*n* = 18)	Nhypokalemia (*n* = 150)	*P* value
Age (years)	46.8 ± 14.6	60.5 (51.8, 68.8)	45.2 ± 13.8	**<0.001**
Sex (male/female)	74/94	9/9	65/85	0.590
BMI	24.7 ± 3.7	24.4 (22.4, 27.0)	24.7 ± 3.9	0.981
Hypertension	55 (32.7%)	9 (50.0%)	46 (30.7%)	0.099
Diabetes	25 (14.9%)	5 (27.8%)	20 (13.3%)	0.202
Incidental tumor	36 (21.4%)	7 (38.9%)	29 (19.3%)	0.108
Sphenoid sinusitis	13 (7.7%)	3 (16.7%)	10 (6.7%)	0.301
Preoperative serum potassium	4.1 ± 0.3	4.1 (3.8, 4.2)	4.1 ± 0.3	0.381
Preoperative serum sodium	140.1 ± 4.8	141.1 (139.0, 142.5)	140.1 ± 4.9	0.979
Preoperative serum calcium	2.3 ± 0.1	2.3 (2.2, 2.4)	2.3 ± 0.1	0.369
Preoperative serum albumin	43.3 ± 3.4	42.4 ± 2.4	43.4 ± 3.5	0.242
Preoperative hyperprolactinemia	77 (45.8%)	7 (38.9%)	70 (46.7%)	0.531
Preoperative high growth hormone	28 (16.7%)	2 (11.1%)	26 (17.3%)	0.738
Preoperative hypogonadism	47 (28.0%)	4 (22.2%)	43 (28.7%)	0.565
Preoperative cortisol hypofunction	19 (11.3%)	3 (16.7%)	16 (10.7%)	0.715
Tumor height (mm)	20.2 ± 11.1	20.2 (17.3, 26.8)	19.9 ± 11.2	0.284
Maximum diameter of tumor (mm)	22.8 ± 10.5	25.3 (19.4, 28.4)	22.4 ± 10.5	0.177
Tumor volume (cm3)	10.7 ± 15.8	7.1 (4.2, 11.0)	10.4 ± 15.2	0.475
Intratumoral cysts or hematoma	65 (38.7%)	6 (33.3%)	59 (39.3%)	0.621
Oppress the optic chiasma	106 (63.1%)	15 (83.3%)	91 (60.7%)	0.060
Knosp grade				0.673
0	29 (17.3%)	1 (5.6%)	28 (18.7%)	
1	39 (23.2%)	4 (22.2%)	35 (23.3%)	
2	38 (22.6%)	4 (22.2%)	34 (22.7%)	
3	37 (22.0%)	5 (27.8%)	32 (21.3%)	
4	25 (14.9%)	4 (22.2%)	21 (14.0%)	
Postoperative hyperprolactinemia	21 (12.5%)	2 (11.1%)	19 (12.7%)	1.000
Postoperative high growth hormone	11 (6.6%)	1 (5.6%)	10 (6.7%)	1.000
Postoperative pituitary-thyroid axis dysfunction	73 (43.5%)	7 (38.9%)	66 (44.0%)	0.679
Postoperative pituitary-gonadal axis dysfunction	67 (39.9%)	9 (50.0%)	58 (38.7%)	0.353
Postoperative cortisol dysfunction	19 (11.3%)	4 (22.2%)	15 (10.0%)	0.249
Postoperative hypoalbuminemia	31 (18.5%)	8 (44.4%)	23 (15.3%)	**0.007**
Serum sodium on the first day after surgery	141.5 ± 3.6	141.0 (138.2, 143.1)	141.6 ± 3.7	0.465
Intraoperative cerebrospinal fluid leaks	33 (19.6%)	5 (27.8%)	28 (18.7%)	0.545
Postoperative DI	80 (47.6%)	10 (55.6%)	70 (46.7%)	0.491
Pathological tumor type				0.344
Non-functional pituitary adenoma	32 (19.1%)	2 (11.1%)	30 (20%)	
GH-pituitary adenoma	19 (11.3%)	0 (0%)	19 (12.7%)	
PRL-pituitary adenoma	33 (19.6%)	1 (5.6%)	32 (21.3%)	
FSH-pituitary adenoma	41 (24.4%)	9 (50.0%)	32 (21.3%)	
ACTH-pituitary adenoma	29 (17.3%)	6 (33.3%)	23 (15.3%)	
TSH-pituitary adenoma	4 (2.4%)	0 (0%)	4 (2.7%)	
Plurihormonal adenomas	10 (6.0%)	0 (0%)	10 (6.7%)	

**Note:**

Values are mean ± standard deviation, median (interquartile range), or number (percentage). DI, diabetes insipidus; GH, growth hormone; PRL, prolactin; FSH, follicle-stimulating hormone; ACTH, adrenocorticotropic hormone; TSH, thyroid-stimulating hormone. Significant *P* values are shown in bold.

Among the hypokalemia group, nine (50.0%) cases had hypertension and five (27.8%) cases had diabetes mellitus. Eight (44.4%) cases developed hypoalbuminemia on the first postoperative day. The mean value of the first hypokalemia episode was 3.05 mEq/L (range 2.77–3.44 mEq/L), occurring on average on the 2.9th postoperative day (range 1st–5th day). The mean lowest serum potassium concentration was 2.85 mEq/L (range 2.69–3.0 mEq/L), with the lowest level occurring on average on the 4th postoperative day (range 2nd–6th day).

### Factors related to postoperative hypokalemia

Results from univariate analysis indicated that age (*P* < 0.001) and hypoalbuminemia on the first postoperative day (*P* = 0.007) exhibited statistically significant differences between the hypokalemia group and the non-hypokalemia group (Table 1). Factors with *P* ≤ 0.1, including age (*P* < 0.001), hypoalbuminemia on the first postoperative day (*P* = 0.007), hypertension (*P* = 0.099), and tumor compressing the optic chiasm (*P* = 0.060), were included in binary logistic regression analysis. The results revealed that age (*OR* 1.09; 95% *CI* [1.03–1.15]; *P* = 0.001) and hypoalbuminemia on the first postoperative day (*OR*, 4.35; 95% *CI* [1.38–13.75]; *P* = 0.012) were independent risk factors for postoperative hypokalemia (Table 2). The optimal cutoff value for age was determined as 50 years through the construction of an age ROC curve (sensitivity 88.9%, specificity 61.3%), with an AUC of 0.792, 95% *CI* [0.70–0.88] (Fig. 1).

**Table 2:** Binary logistic regression analysis of the risk of postoperative hypokalemia.

Factors		*OR*		95% *CI*		*P* Value
Age		1.09		[1.03–1.15]		**0.001**
Postoperative hypoalbuminemia		4.35		[1.38–13.75]		**0.012**
Hypertension		0.72		[0.21–2.50]		0.610
Oppress the optic chiasma		1.62		[0.41–6.44]		0.493

**Note:**

OR, odds ratio; CI, confidence interval; DI, diabetes insipidus; Significant *P* values are shown in bold.

## Discussion

Currently, endoscopic pituitary tumor resection surgery stands as the most mainstream approach for pituitary tumor surgery (Nieman et al., 2015), offering numerous advantages such as close observation, clear illumination, minimally invasive techniques, reduced trauma, and shorter hospital stays. However, this procedure also comes with certain complications, with postoperative hypokalemia being one of the most common. Mild hypokalemia usually presents with no obvious symptoms or atypical manifestations such as headache, dizziness, nausea, vomiting, and constipation, which generally do not significantly impact patients. Studies indicate that symptoms typically appear when serum potassium levels fall below 3.0 mEq/L (Kardalas et al., 2018). Severe hypokalemia can lead to multi-organ dysfunction, including hypokalemic periodic paralysis, paralytic ileus, arrhythmias, respiratory failure, and even death (Gheeraert et al., 2006; Weir & Espaillat, 2015). The incidence of perioperative hypokalemia in patients is approximately 14–40% (Krogager et al., 2021; Nilsson et al., 2017), highlighting the importance of physician vigilance. Nevertheless, research on post-endoscopic pituitary tumor resection hypokalemia remains insufficient. Therefore, we aim to explore related risk factors through a retrospective cohort study to provide references for preventing postoperative hypokalemia after endoscopic pituitary tumor resection.

Among 168 patients, 18 (10.7%) developed postoperative hypokalemia. Due to few factors with *P* < 0.05, factors with *P* ≤ 0.1 were included in binary logistic regression analysis following univariate analysis. The results indicated that age (*OR*, 1.09; 95% *CI* [1.03–1.15]; *P* = 0.001) and hypoalbuminemia on the first postoperative day (*OR*, 4.35; 95% *CI* [1.38–13.75]; *P* = 0.012) were independent risk factors for postoperative hypokalemia. Given the retrospective nature of this research, the sample size of 168 is relatively modest and may predispose to type II errors. The data collection process might also have introduced confounding variables that could influence the reliability of our findings. Consequently, the analysis was confined to a limited dataset from a single center, rendering the conclusions primarily informative but not definitive. Future studies employing larger or multicenter cohorts are necessary to validate our results.

The mechanism of postoperative hypokalemia following endoscopic pituitary tumor resection is not fully understood. Through a retrospective study, Li et al. (2021) found significant associations between age, PA type, weight, APTT, urea, EOS, and PCT with postoperative hypokalemia. Age exhibited a significantly increased risk for postoperative hypokalemia (*OR*, 1.06; 95% *CI* [1.02–1.10]; *P* = 0.002), which aligns with our study findings. You et al.’s (2017) study suggested that ACTH-secreting pituitary adenoma is an independent risk factor for postoperative hypokalemia (*OR*, 4.92; 95% *CI* [1.18–20.48]; *P* = 0.029), suggesting immediate potassium supplementation postoperatively for such patients to prevent hypokalemia (You et al., 2017). However, in our current study, we identified age ≥50 years and postoperative hypoalbuminemia on the first day as independent risk factors for postoperative hypokalemia. It is important to note that retrospective studies have inherent limitations such as bias and confounding effects. The disparities in research findings between our study and You et al.’s (2017) study may be attributed to differences in sample size, data collection methods, methodology, and statistical analysis techniques. Given the limited research available on postoperative hypokalemia in pituitary tumors, we have only compared our findings with one previous study at this time. Additional research is needed to further explore this topic and validate our results.

Furthermore, TSH-secreting pituitary adenomas can induce thyroid hormone secretion. Thyroid hormones enhance Na-K pump activity on cell membranes, promoting potassium influx into cells. Therefore, TSH-secreting pituitary adenomas can lead to hypokalemia or even thyrotoxic periodic paralysis (TPP) due to increased thyroid hormone levels (Pappa et al., 2010; Hsu et al., 2003). TSH/ACTH-secreting tumors often elevate intra-patient TSH, ACTH, thyroid hormone, and cortisol levels, ultimately lowering blood potassium levels. Some speculate that tumor tissue excision during surgery results in hormone release into the blood from normal and stimulated pituitary cells, mediating hormone-induced hypokalemia (You et al., 2017). However, this hypothesis remains unconfirmed. Others propose that traction on the pituitary stalk during surgery increases ADH release, leading to water and sodium retention (Mo et al., 2024), subsequently reducing serum potassium concentrations and causing hypokalemia. Our research indicates that age and postoperative hypoalbuminemia are independent risk factors for postoperative hypokalemia. The synthesis and release of antidiuretic hormone ADH are influenced by several factors including plasma osmotic pressure, neurotransmitter activity, and central nervous system regulation. In elderly patients, postoperative stress can activate the central nervous system, elevating ADH levels, which in turn increases renal water reabsorption and promotes water retention. This process can lead to dilutional hypokalemia. Additionally, serum albumin plays multiple roles in regulating potassium ion concentrations. Hypoalbuminemia can decrease plasma osmotic pressure, disrupting fluid equilibrium across cellular membranes. When the intracellular osmotic pressure surpasses extracellular pressure, water and ions, including potassium, migrate into cells, reducing extracellular potassium levels. Given that albumin carries a negative charge, its reduced levels can disturb the ion balance, affecting potassium ion dynamics. Normally, potassium ions predominantly exist in a free state within the plasma. However, lower albumin levels can induce binding of potassium ions with other substances, resulting in a decreased concentration of free potassium ions in the plasma. Studies also suggest that decreased skeletal muscle mass and increased comorbidities in elderly individuals make them more susceptible to hypokalemia (Abensur Vuillaume et al., 2020; Bardak et al., 2017; Kojima, Mizokami & Akishita, 2020).

The majority of potassium ions are distributed intra-cellularly, with a small fraction being extracellular (Weir & Espaillat, 2015). The serum potassium ion concentration is influenced by various factors, such as neuro-humoral, medications, diseases, *etc*. Insulin, catecholamines, thyroid hormones, and aldosterone can all cause hypokalemia (Kamel, Schreiber & Halperin, 2018; DuBose, 2017). Aldosterone primarily lowers blood potassium levels by increasing the renal excretion of potassium ions (Unwin, Luft & Shirley, 2011). Insulin and catecholamines, on the other hand, increase cellular uptake of potassium by activating the Na-K pump, leading to hypokalemia (Unwin, Luft & Shirley, 2011; Ewart & Klip, 1995). Thyroid hormones can stimulate the synthesis of the Na-K pump to reduce serum potassium ion concentration. This is consistent with some research findings: TSH-secreting pituitary adenomas can lead to thyrotoxic periodic paralysis, although the incidence is very low (Pappa et al., 2010; Hsu et al., 2003). However, in our study, no clear correlation was found between TSH-secreting pituitary adenomas and postoperative hypokalemia, indicating the need for further research to establish their relationship. Typically, most cases of hypokalemia in clinical practice are drug-induced (Crop et al., 2007). The main mechanisms through which drugs induce hypokalemia are increasing intracellular transport of potassium ions or increasing potassium ion consumption. Commonly used drugs such as loop diuretics, penicillins, aminoglycoside antibiotics, laxatives, and enemas can all lead to potassium loss, resulting in decreased total body potassium levels and hypokalemia (Unwin, Luft & Shirley, 2011; Unwin et al., 2000; Gill, DuBe & Young, 1977). β2-agonists and calcium channel blockers can increase potassium transport into cells, affecting potassium distribution and causing hypokalemia (Minella & Schulman, 1991; Braden et al., 1997; Bremner et al., 1992). Certain diseases can also lead to hypokalemia, such as leukemia, primary hyperaldosteronism, diabetic patients treated with insulin, and patients with diarrhea. Hypokalemia is a common electrolyte disturbance following surgery, closely linked to acid-base balance, water balance, renal function, and kidney disease. In the early postoperative period, patients often experience fluid retention, leading to dilutional hypokalemia. This mechanism is primarily attributed to surgical stress inducing a state of stress in patients, stimulating the central nervous system, increasing the synthesis and release of ADH, enhancing water reabsorption, and causing dilutional hypokalemia. Furthermore, patients with stable pituitary tumors typically resume their normal diet on the first day after surgery. However, nasal surgery can result in discomfort and airway obstruction in the nose and throat during the early postoperative period, impacting appetite and reducing food intake. This can increase the risk of hypokalemia due to dietary factors. In patients experiencing metabolic alkalosis postoperatively, there is an exchange of H-K ions during compensation processes which leads to a decrease in extracellular K ions. While the kidneys normally play a role in potassium excretion, early postoperatively potassium ions often exhibit negative balance. Patients with kidney diseases such as chronic kidney disease or acute kidney injury may experience decreased renal potassium excretion. In these cases, if postoperative hypokalemia occurs, serum potassium levels may not be low or normal during testing; they may even be higher than normal levels. Therefore, when studying postoperative hypokalemia in patients with kidney diseases, it is crucial to consider the complex influence of kidneys on potassium ions.

Most patients with hypokalemia do not show obvious clinical symptoms and are usually identified through routine serum electrolyte testing. However, for patients with comorbid conditions such as hypertension, diabetes, heart failure, or kidney disease, hypokalemia can lead to adverse clinical outcomes (Gennari, 1998; Cohn et al., 2000). Therefore, proactive management is necessary for postoperative hypokalemia patients. For safety reasons, our center primarily uses oral potassium supplementation with intravenous supplementation as secondary. It is important to note that before administering potassium, it is often necessary to rule out drug-induced or underlying disease-related hypokalemia. For example, in heart failure patients requiring diuretic therapy, a switch to potassium-conserving diuretics may be considered. Oral potassium supplements can be achieved through dietary and medication intake. For dietary supplementation, encouraging patients to consume potassium-rich foods is common practice. As for medication supplementation, potassium chloride solution or tablets are typically used, with a recommended dosage of 10–15 ml of potassium chloride solution three times a day or 2–3 tablets of potassium chloride three times a day. However, research suggests that oral potassium chloride tablets may increase the risk of gastrointestinal bleeding (Gennari, 1998), which is why our center primarily uses potassium chloride solution for medication supplementation. After administering potassium supplementation, blood potassium levels are monitored to determine the need for continued supplementation. In cases of severe hypokalemia with or without significant clinical symptoms, intravenous potassium supplementation may be necessary. A common regimen is administering 1.5 g potassium chloride in 500 ml 5% sodium chloride intravenously, with a recommended infusion rate not exceeding 20 mmol/h. Patients receiving intravenous potassium should undergo continuous cardiac monitoring, with particular attention to ECG changes. Currently, there are no definitive guidelines on the duration of potassium supplementation. Literature suggests that supplementation may be required for several days to weeks (Weir & Espaillat, 2015; Cohn et al., 2000). Over 50% of clinically significant cases of hypokalemia are associated with hypomagnesemia, which can complicate the treatment of hypokalemia (Huang & Kuo, 2007). Therefore, scholars propose considering appropriate magnesium supplementation for refractory hypokalemia1 (Weir & Espaillat, 2015). According to Kardalas et al. (2018), oral potassium supplementation may suffice when blood potassium levels range from 2.5–3.0 mEq/L. Intravenous potassium supplementation is recommended when blood potassium levels are below 2.5 mEq/L, along with ECG monitoring and regular blood potassium checks. Hypokalemia has diverse etiologies, and the related pathophysiological mechanisms are complex. Further multi-center retrospective studies are needed to identify more robust and convincing risk factors, while additional basic research is required to confirm the underlying mechanisms of onset.

In our study, our objective was to investigate the correlation between postoperative hypokalemia following endoscopic pituitary tumor surgery and patient clinical outcomes. The results may not have demonstrated a significantly higher incidence of hypokalemia in pituitary surgery patients compared to other clinical patients, possibly due to our categorization method. We classified patients with postoperative serum potassium ion concentrations <3.0 mEq/L into the postoperative hypokalemia group, selecting the lowest value if multiple tests showed serum potassium ion concentration <3.0 mEq/L. Previous studies have extensively discussed postoperative hypokalemia, including all patients with postoperative serum potassium ion concentrations <3.5 mEq/L, which may encompass individuals without true hypokalemia, such as asymptomatic individuals with serum potassium levels at 3.4 mEq/L. Isolated postoperative serum potassium levels of 3.4 mEq/L are common and typically do not indicate postoperative complications.

Therefore, recognizing the symptoms and risk factors of hypokalemic patients requiring prompt intervention after surgery can equip clinicians with improved diagnostic and treatment strategies.

## Conclusions

The mechanism of postoperative hypokalemia in pituitary tumors following endoscopic surgery is not fully understood. We believe that patients aged ≥50 years and those with postoperative hypoalbuminemia on the first day are at a higher risk of developing hypokalemia postoperatively. Therefore, such patients should undergo intensified electrolyte monitoring postoperatively, with particular emphasis on proactive prevention of hypokalemia between the 4th and 5th day post-surgery.

## Supplemental Information

10.7717/peerj.18536/supp-1 Supplemental Information 1 Raw data.

## Additional Information and Declarations

### Data Availability

The following information was supplied regarding data availability: The raw measurements are available in the Supplemental File.
